# Relationship between frequency of hypoglycemic episodes and changes in carotid atherosclerosis in insulin-treated patients with type 2 diabetes mellitus

**DOI:** 10.1038/srep39965

**Published:** 2017-01-09

**Authors:** Tomoya Mita, Naoto Katakami, Toshihiko Shiraiwa, Hidenori Yoshii, Nobuichi Kuribayashi, Takeshi Osonoi, Hideaki Kaneto, Keisuke Kosugi, Yutaka Umayahara, Masahiko Gosho, Iichiro Shimomura, Hirotaka Watada

**Affiliations:** 1 Department of Metabolism & Endocrinology, Juntendo University Graduate School of Medicine, Hongo 2-1-1, Bunkyo-ku, Tokyo 113-8421, Japan; 2 Department of Metabolic Medicine, Osaka University Graduate School of Medicine, 2-2, Yamadaoka, Suita, Osaka 565-0871, Japan; 3 Department of Metabolism and Atherosclerosis, Osaka University Graduate School of Medicine, 2-2, Yamadaoka, Suita, Osaka 565-0871, Japan; 4 Shiraiwa Medical Clinic, 4-10-24 Houzenji, Kashiwara, Osaka 582-0005, Japan; 5 Department of Medicine, Diabetology & Endocrinology, Juntendo Tokyo Koto Geriatric Medical Center, Shinsuna 3-3-20, Koto-ku, Tokyo 136-0075, Japan; 6 Misaki Naika Clinic, 6-44-9 Futawahigashi, Funabashi, Chiba 274-0805, Japan; 7 Naka Memorial Clinic, 745-5, Nakadai, Naka City, Ibaraki 311-0113, Japan; 8 Osaka Police Hospital, 10-31 Kitayamacho, Tennoji-ku, Osaka 543-0035, Japan; 9 Osaka General Medical Center, 3-1-56 Bandai-Higashi, Sumiyoshi-ku, Osaka 558-8558, Japan; 10 Department of Clinical Trial and Clinical Epidemiology, Faculty of Medicine, University of Tsukuba, 1-1-1, Tennodai, Tsukuba, Ibaraki 305-8575, Japan

## Abstract

The effect of hypoglycemia on the progression of atherosclerosis in patients with type 2 diabetes mellitus (T2DM) remains largely unknown. This is a post hoc analysis of a randomized trial to investigate the relationship between hypoglycemic episodes and changes in carotid intima-media thickness (IMT). Among 274 study subjects, 104 patients experienced hypoglycemic episodes. Increases in the mean IMT and left maximum IMT of the common carotid arteries (CCA) were significantly greater in patients with hypoglycemia compared to those without hypoglycemia. Classification of the patients into three groups according to the frequency of hypoglycemic episodes showed that high frequency of hypoglycemic events was associated with increases in mean IMT-CCA, and left max-IMT-CCA and right max-IMT-CCA. In addition, repetitive episodes of hypoglycemia were associated with a reduction in the beneficial effects of sitagliptin on carotid IMT. Our data suggest that frequency of hypoglycemic episodes was associated with changes in carotid atherosclerosis.

While type 2 diabetes mellitus (T2DM) is a risk factor for cardiovascular disease (CVD), which is one of the major causes of morbidity and mortality in these patients^1^, large randomized clinical trials did not show the benefits of strict glycemic control on CVD in patients with established atherosclerosis or longstanding T2DM^2^,^3^,^4^. On the other hand, a recent study reported that the occurrence of hypoglycemia was associated with increased risk of CVD and all-cause mortality in insulin-treated patients with type 1 diabetes mellitus (T1DM) and T2DM^5^.

Hypoglycemia is a common adverse effect of management for diabetes, especially insulin therapy, and a barrier to optimal glycemic control. Hypoglycemia affects blood constituents^6^,^7^, inflammatory cytokine levels^8^,^9^, and coagulation and fibrinolysis factors^10^,^11^, all of which might promote the progression of atherosclerosis. Indeed, the acute effects of hypoglycemia, such as sympatho-adrenal activation, catecholamine release on inflammation, endothelial injury, and pro-atherothrombotic biomarkers^12^,^13^, are well known in patients with T1DM. Also, a cross sectional study demonstrated that repeated episodes of hypoglycemia were associated with preclinical atherosclerosis evaluated by carotid and femoral echography and measurement of flow-mediated brachial dilatation in patients with T1DM^14^. However, the long-term effect of hypoglycemia on the progression of atherosclerosis remains largely unknown in patients with T2DM.

Sitagliptin, a dipeptidyl peptidase-4 (DPP-4) inhibitor, improves glycemic control without increasing the risk of hypoglycemia^15^,^16^. Recently, we reported that sitagliptin treatment attenuated the increases in carotid intima-media thickness (IMT) in insulin-treated patients with T2DM compared with the conventional treatment^17^. In the same study, we demonstrated that sitagliptin treatment was superior to conventional treatment in terms of HbA1c reduction without increasing the incidence of hypoglycemic episodes, consistent with previous studies^18^,^19^,^20^. However, we have not investigated whether hypoglycemic events are associated with a reduction in the beneficial effects of sitagliptin on the changes in carotid atherosclerosis.

The aim of the present post hoc-analysis was to investigate the relationship between hypoglycemic events and changes in carotid IMT in insulin-treated patients with T2DM.

## Results

### Participants

In the original study, 104 of 274 patients experienced hypoglycemia (52 of the sitagliptin group and 52 of the conventional group) over the 104 week follow-up. There was no significant differences in the mean number of hypoglycemic events between the two groups (0.34 ± 0.85 episodes/month/person in the sitagliptin group vs 0.36 ± 0.80 in the conventional group). One episode of severe hypoglycemic event occurred in each group. Subgroup analysis according to the occurrence of hypoglycemia showed that patients with hypoglycemia were older, had lower estimated glomerular filtration rate (eGFR) level, lower C-peptide level and had more frequent use of sulfonylurea and α-glucosidase inhibitors, but were more lean, less likely to be smokers, had lower HbA1c, total-cholesterol, triglyceride, hs-CRP (high-sensitivity C-reactive protein) and interleulin-6, and few use of glinides (Table 1).

### Occurrence of hypoglycemia is associated with the changes in carotid IMT

Of the 274 patients in the original study, 243 patients with available carotid IMT data at baseline and 104 weeks were included in this post hoc analysis. Among them, 98 patients experienced hypoglycemia (49 of the sitagliptin group and 49 of the conventional group). In the analysis of covariance models that included the occurrence of hypoglycemia, age, gender, baseline IMT and the original treatment group (model 1), the changes in mean IMT of the common carotid arteries (mean-IMT-CCA) and left maximum IMT of the common carotid artery (max-IMT-CCA), but not right max-IMT-CCA, were significantly higher in patients with hypoglycemia than those without (Table 2). Similar findings were noted even in the adjusted models, including model 2 (model 1+ body mass index (BMI) + current smoking), model 3 (model 2 + HbA1c, total cholesterol, high density lipoprotein-cholesterol, triglyceride and systolic blood pressure), model 4 (model 3+ eGFR + angiotensin-converting enzyme inhibitors/angiotensin II receptor blocker, statins and anti-platelets), model 5 (model 4 + sulfonylurea + glinides + α-glucosidase inhibitors), and model 6 (model 5 + C-peptide + hsCRP + interleukin 6).

Next, we performed subgroup analysis according to type of treatment. As shown in Table 3, the mean-IMT-CCA was significantly increased relative to baseline in patients with hypoglycemia, but not in those without hypoglycemia in the conventional group. In addition, the changes in mean-IMT-CCA and left max-IMT-CCA tended to be greater in patients with hypoglycemia compared to those without. More importantly, in patients without hypoglycemia, the changes in mean-IMT-CCA and left max-IMT-CCA, but not right max-IMT-CCA, were significantly smaller in the sitagliptin treatment group than those in the conventional treatment group, while these findings were not observed in patients with hypoglycemia (Table 3). These data showed that hypoglycemia was associated with a reduction in the beneficial effects of sitagliptin.

### Frequency of hypoglycemia is associated with changes in carotid IMT

**Figure 1:**
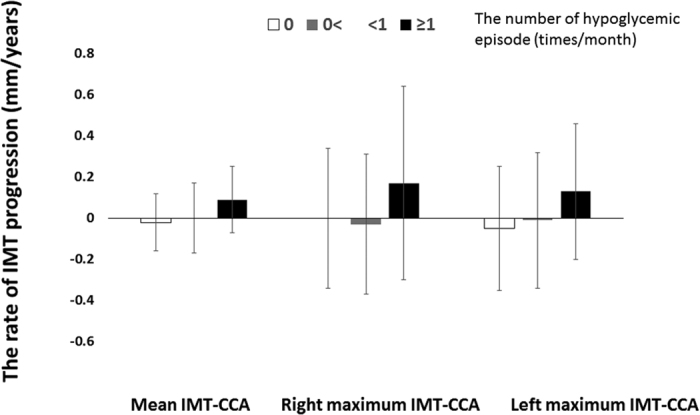
Changes in IMT according to the frequency of hypoglycemic episodes. Data are mean ± SD.

Next, to investigate the relationship between the frequency of hypoglycemia and changes in carotid IMT, we divided patients with the occurrence of hypoglycemia into three groups; patients without hypoglycemia, patients with less than 1 times/month hypoglycemia, and patients with more than 1 times/month hypoglycemia. Trend associations across three groups and changes in IMT were evaluated by univariate and multivariate linear regression analyses (Table 4). The frequency of hypoglycemia was associated with changes in mean-IMT-CCA and left max-IMT-CCA, but not right max-IMT-CCA in unadjusted model (model 1). Almost similar findings were noted even in the adjusted models, including model 2 (model 1 + baseline IMT), model 3 (model 2+ age + gender + the original treatment group), model 4 (model 3+ BMI + current smoking), model 5 (model 4 + HbA1c, total cholesterol, high density lipoprotein-cholesterol, triglyceride and systolic blood pressure), model 6 (model 5 + eGFR + angiotensin-converting enzyme inhibitors/angiotensin II receptor blockers, statins and anti-platelets), model 7 (model 6 + sulfonylurea + glinides + α-glucosidase inhibitors), and model 8 (model 7 + C-peptide + hsCRP + interleukin 6). In addition, analysis of data of the entire population showed that increased frequency of hypoglycemia was associated with changes in mean-IMT-CCA (P for difference among the three groups = 0.002), right max-IMT-CCA (P = 0.032), and left max-IMT-CCA (P = 0.02) (Fig. 1).

### Changes in other parameters

The change in HbA1c (value at end of study - value at baseline) was comparable between the hypoglycemia group (−0.2 ± 0.9%) and no-hypoglycemia group (−0.4 ± 1.1%) (Supplementary Table 1). Similarly, there were no differences in changes in other risk factors for atherosclerosis such as hypertension and lipid parameters during the observation period (Supplementary Table 1).

## Discussion

Hypoglycemia is a common adverse effect of insulin treatment for T2DM^21^. Previous studies demonstrated that the frequency of hypoglycemic episodes remains unacceptably high even when DPP-4 inhibitors are added to insulin therapy, although they did not increase the incidence of hypoglycemic episodes^18^,^19^,^20^. The same appears to be true in the present study. Indeed, the high frequency of hypoglycemic events is associated with increases in carotid atherosclerosis. Previous studies identified several factors, such as BP, BMI^22^ and lipid parameters^23^, but not hypoglycemic events, to be associated with the changes in carotid artery atherosclerosis in patients with T2DM. Thus, this was the first study to demonstrate that frequent episodes of hypoglycemia are associated with changes in carotid artery atherosclerosis in insulin-treated patients with T2DM.

While most clinical data showing associations between hypoglycemia and adverse CV risk/mortality risk have derived from “serious hypoglycemic” episodes^24^,^25^,^26^, the effect of mild hypoglycemia on adverse CV outcomes remains still unknown. On the other hand, virtually, this study showed the relation between frequency of mild hypoglycemia and changes in IMT because most of the hypoglycemic episodes were mild. In this regard, further prospective large sample size studies are required to address whether mild hypoglycemia is also associated with adverse CV risk/mortality risk

In this study, patients with hypoglycemia were older, suffered more severe renal dysfunction, had less endogenous insulin and more frequent use of sulfonylurea. Thus, it is possible that hypoglycemia is only a maker of patients with advanced stages of T2DM and/or those prone to CVD. However, the occurrence of hypoglycemia was still associated with the changes in carotid IMT even after adjustment for these confounders. Furthermore, the control of atherosclerosis risk factors was not consistently worse in patients with the occurrence of hypoglycemia because they were less obese, were less likely to be smokers, achieved better glycemic and lipid controls and had lower serum levels of inflammatory cytokines, reflecting patients who were at lower risk for CVD. Indeed, almost similar findings were observed in analysis of covariance models after adjustment for those confounding factors. These data support that hypoglycemia by itself seem to be a contributing factor for atherosclerosis.

The mechanism of the deleterious effect of hypoglycemia on carotid vascular wall may be multidimensional and possibly involves changes in blood constituents^6^,^7^, inflammation^8^,^9^, and coagulation and fibrinolysis^10^,^11^, through counter-regulatory defense responses to hypoglycemia. While these responses and changes are transient and play a crucial role in protecting vital organs, previous studies demonstrated that acute insulin-induced hypoglycemia can provoke inflammatory response, platelet aggregation and endothelial dysfunction in patients with T1DM^12^,^27^. Thus, the progression of atherosclerosis may be accelerated if hypoglycemic episodes occur frequently. However, repeated episodes of relatively mild hypoglycemia have been shown to reduce the counter-regulatory defense responses to hypoglycemia in intensive treatment of T2DM^28^. Nevertheless, at least in Goto-Kakizaki rats, diabetic model rats, these responses never disappear and enhance monocyte adhesion to endothelial cells in the aorta, leading to increased intimal thickening^29^. In agreement with this finding, our data demonstrated that changes in carotid atherosclerosis increased with increased number of hypoglycemic events.

What is the precise mechanism through which repeated episodes of hypoglycemia promote the progression of atherosclerosis? In the present study, we did not find any association between serum biomarkers of inflammation or endothelial injury and frequency of hypoglycemia or carotid IMT changes (data not shown). It is possible that this negative relationship may be due to several limiting features of such biomarkers. Indeed, the amount of such biomarkers is known to be affected by both transient and chronic non-atherosclerotic diseases and drug treatment within a relatively short time^30^. In the future, it would be interesting to determine whether repeated episodes of hypoglycemia could affect plaque characteristics of the carotid arteries evaluated by sophisticated studies such as Computed Tomography and Magnetic Resonance Imaging. These studies might shed light on pro-inflammatory effects of repetitive hypoglycemia on the vasculature. Another possibility is that hypoglycemia directly induced hypercatecholaminemia by acting on the vascular wall^29^,^31^. However, it is difficult to evaluate serum catecholamine levels during hypoglycemia in clinical setting. Further studies are required to clarify the mechanism of the influence of hypoglycemia on the vasculature.

It is reported that glucagon-like peptide-1 (GLP-1) attenuates hypoglycemia-induced oxidative stress, inflammation and endothelial dysfunction in patients with T1DM^27^. Therefore, there is a great interest in determining whether DPP-4 inhibitors play an important role in protecting against hypoglycemia-induced vascular damages. In this regard, our data suggest that repetitive episodes of hypoglycemia are associated with a reduction in the beneficial effects of sitagliptin on the changes in carotid atherosclerosis. The different results may be due to the different subjects (T1DM vs. T2DM) or different levels of GLP-1 (DPP-4 inhibitor vs. GLP-1). In any case, when DPP-4 inhibitors are used as add-on therapy to insulin therapy, one should take precaution against potential hypoglycemia by, for example, reducing the dose of insulin.

### Study limitations

The present study has certain limitations. First, this was a post hoc and exploratory analysis of a randomized unblinded trial and the sample size was relatively small. The analysis is limited by the study design and this make the significance of the findings less clear. Thus, the results should be interpreted with caution. Second, we evaluated self-reported hypoglycemic events through self-monitoring of blood glucose (SMBG) levels and hypoglycemic symptoms. Thus, there may be a risk of underestimation of the incidence of hypoglycemic episodes. In an effort to overcome possible recall bias, we confirmed the occurrence of hypoglycemia at each visit of each patient. Third, we did not evaluate hypoglycemia awareness status. Since some episodes of non-severe hypoglycemia could be asymptomatic, they were unreported by patients. Thus, we could not rule out possible underestimation of the deleterious effects of hypoglycemia on carotid atherosclerosis. Fourth, there were some discrepancies in the results of carotid IMT although the results showed a similar pattern. These differences may be due to the underpowered sample and side difference in IMT-CCA^32^.

## Conclusions

Our data suggest that frequent episodes of hypoglycemia were associated with changes in carotid atherosclerosis in insulin-treated patients with T2DM. Physicians need to take care in avoiding the occurrence of hypoglycemic episodes in the clinical setting in order to minimize the process of atherosclerogenesis.

## Methods

### Study population

We performed a post hoc analysis from the Sitagliptin Preventive study of Intima-media thickness Evaluation (SPIKE). The study design, inclusion and exclusion criteria, study schedule and measurements were described in detail previously^17^,^33^. Briefly, insulin-treated Japanese T2DM patients free of past history of apparent CVD who periodically attended the Outpatient Diabetes Clinics at 12 centers across Japan were asked to participate in this study. A total of 282 participants were randomly allocated to either the sitagliptin group (n = 142) or the conventional treatment group (using drugs other than the DPP-4 inhibitor) (n = 140). Finally, 137 in the sitagliptin group and 137 in the conventional treatment group were included in the full analysis set. Each participant underwent ultrasonography of the carotid arteries including mean-IMT-CCA and in the right and left max-IMT-CCA performed by expert sonographers at the start of the study, and the procedure was repeated after 52 and 104 weeks.

All patients who agreed to participate were entered into the study. The protocol was approved by the Institutional Review Board of each participating institution (Juntendo University Graduate School of Medicine, Osaka University Graduate School of Medicine, Juntendo Tokyo Koto Geriatric Medical Center, Naka Memorial Clinic, Osaka Police Hospital, Osaka General Medical Center, Kansai Rosai Hospital, Sasebo Chuo Hospital, Jiyugaoka Medical Clinic, Ikeda Municipal Hospital) in compliance with the Declaration of Helsinki and current legal regulations in Japan. Written informed consent was obtained from all the participants after full explanation of the study. This study has been registered on the University Hospital Medical Information Network Clinical Trials Registry, which is a non-profit organization in Japan and meets the requirements of the International Committee of Medical Journal Editors (UMIN000007396).

### Definition of hypoglycemia

All adverse events including hypoglycemia were recorded at each visit during study, as described previously^17^. During the study, participants were asked to submit their records of SMBG and whether they had a sign and symptoms of hypoglycemia. Hypoglycemia was defined based on confirmation by measurement of plasma glucose of ≤3.9 mmol/L and/or self-reported probable hypoglycemic symptom. Severe hypoglycemia was defined as events requiring aid of another person to administer treatment.

### Measurement of carotid IMT

Ultrasonographic scans of the carotid artery were performed by expert sonographers who were specifically trained to perform the prescribed study examination, as reported previously^17^,^33^. To avoid inter-sonographer variability, each participant was examined by the same sonographer with the same equipment throughout all the visits. To avoid inter-reader variability, all scans were electronically stored and emailed to the central office (IMT Evaluation Committee, Osaka, Japan) to be read by a single experienced reader blinded to the clinical features of the patients, in a random order, using automated digital edge-detection software (Intimascope; MediaCross, Tokyo, Japan)^17^,^33^. The software system averages 200 points of IMT values in the segment 2 cm proximal to the dilation of the carotid bulb (mean-IMT-CCA). In addition, the greatest thicknesses of IMT, including plaque lesions in the common carotid arteries (max-IMT-CCA) were also measured separately. The reproducibility of IMT measurement was very high as described previously^17^.

### Statistical analysis

Data were reported as mean ± SD. Statistical analysis was performed using analysis of covariance models that included several variables in addition to occurrence of hypoglycemia, such as age, gender and baseline IMT (see Table 2 for details). Baseline and follow-up group comparisons were assessed with the Student’s t-test or Wilcoxon’s rank sum test for continuous variables and Fisher’s exact test for categorical variables. Changes from baseline to treatment visits were assessed with one-sample t-test and Wilcoxon’s signed rank test within the group. We categorized patients into the following three groups according to the number of hypoglycemic episodes during follow-up: 1) those with no episodes, 2) those with less than one episode per month, and 3) those with one or more episodes. Comparisons among the three groups were performed by one-way analysis of variance. Trend associations across the three groups and changes in IMT were evaluated by univariate and multivariate linear regression analyses. All statistical tests were two-sided with 5% significance level. All analyses were performed using the SAS software version 9.4 (SAS Institute, Cary, NC).

## Additional Information

**How to cite this article**: Mita, T. *et al*. Relationship between frequency of hypoglycemic episodes and changes in carotid atherosclerosis in insulin-treated patients with type 2 diabetes mellitus. *Sci. Rep.* **7**, 39965; doi: 10.1038/srep39965 (2017).

**Publisher's note:** Springer Nature remains neutral with regard to jurisdictional claims in published maps and institutional affiliations.

## Figures and Tables

**Table 1:** Clinical characteristics of patients of the two groups.

Parameters	Hypoglycemia group (n = 104)	No-Hypoglycemia group (n = 170)	P value
Mean number of hypoglycemic events (n/month/person)	0.93 ± 1.13	0.00 ± 0.00	<0.001
Age (years)	65.6 ± 9.4	62.6 ± 10.0	0.014
Gender (males) (%)	59 (57)	106 (62)	0.38
Body Mass Index	24.3 ± 3.7	25.5 ± 4.2	0.021
Systolic blood pressure (mmHg)	130 ± 15	132 ± 15	0.24
Diastolic blood pressure (mmHg)	74 ± 10	75 ± 12	0.32
Current smoking	30 (22)	29 (21)	0.22
Hypertension	60 (58)	101 (59)	0.80
Dyslipidemia	61 (59)	114 (67)	0.19
Duration of diabetes (years)	18.6 ± 8.5	16.5 ± 8.5	0.053
HbA1c (%)	7.8 ± 0.8	8.2 ± 1.1	0.011
HbA1c (mmol/mol)	62.2 ± 8.9	65.8 ± 12.3	0.011
C-peptide (ng/ml)	0.32 ± 0.24	0.44 ± 0.25	<0.001
Total cholesterol (mmol/l)	4.79 ± 0.76	5.10 ± 0.94	0.005
LDL cholesterol (mmol/l)	2.67 ± 0.66	2.90 ± 0.78	0.014
HDL cholesterol (mmol/l)	1.47 ± 0.42	1.39 ± 0.35	0.09
Triglyceride (mmol/l)	1.04 (0.69, 1.69)	1.21 (0.97, 1.74)	0.008
eGFR (mL/min/1.73 m^2^)	73.9 ± 17.6	81.7 ± 24.9	0.006
UAE (mg/g creatinine)	18.7 (7.1, 59.8)	19.7 (9.3, 88.2)	0.52
hsCRP (ng/dl)	380 (209, 911)	523 (264, 1340)	0.042
IL-6 (ng/dl)	1.5 (1.1, 2.2)	2.1 (1.4, 3.4)	<0.001
ICAM-1 (ng/ml)	229 (193, 278)	228 (181, 265)	0.59
VCAM-1 (ng/ml)	736 (634, 920)	774 (662, 977)	0.22
Mean IMT-CCA (mm)	0.85 ± 0.20	0.83 ± 0.19	0.35
Right maximum IMT-CCA (mm)	1.10 ± 0.36	1.02 ± 0.34	0.073
Left maximum IMT-CCA (mm)	1.11 ± 0.35	1.10 ± 0.38	0.82
Use of oral glucose-lowering agents			
Metformin	41 (39)	56 (33)	0.3
Sulfonylurea	21 (20)	11 (6)	<0.001
Glinides	1(1)	20 (12)	<0.001
Thiazolidinediones	9 (9)	15 (9)	1.00
α-glucosidase inhibitors	44 (42)	39 (23)	0.001
Use of anti-hypertensive agents			
Angiotensin-converting enzyme inhibitors	4 (4)	8 (5)	1.00
Angiotensin II receptor blockers	50 (48)	72 (42)	0.38
Calcium channel blocker	30 (29)	54 (32)	0.69
Diuretic drugs	10 (10)	15 (9)	0.83
α-adrenergic receptor antagonist	2 (2)	2 (1)	0.64
β-adrenergic receptor antagonist	2 (1)	1 (1)	0.56
Use of lipid-lowering agents			
Statins	46 (44)	83 (49)	0.53
Ezetimibe	8 (8)	6 (4)	0.16
Fibrates	3 (3)	4 (2)	1.00
Use of anti-thrombotic agents			
Antiplatelet agents	23 (22)	36 (21)	0.88

Data are number (%) of patients or mean ± SD values or median (range) values. Differences in parameters between groups were analyzed by the Student’s t-test or Wilcoxon’s rank sum test for continuous variables and Fisher’s exact test for categorical variables. CCA, common carotid artery; estimated glomerular filtration rate; ICAM-1, intercellular adhesion molecule 1; IMT, intima-media thickness; ICAM-1, intercellular adhesion molecule 1; VCAM-1, vascular cell adhesion molecule 1; IL-6, interleukin 6; hs-CRP, high-sensitivity C-reactive protein; UAE, urinary albumin excretion.

**Table 2:** Changes in intima-media thickness from baseline by analysis of covariance models.

	Hypoglycemia group	No-Hypoglycemia group	Adjusted mean difference between groups	P value
Mean IMT-CCA (mean change from baseline; SE)				
Model 1	0.027 (0.015)	−0.023 (0.012)	−0.050 (−0.088, −0.013)	0.009
Model 2	0.027 (0.015)	−0.023 (0.012)	−0.050 (−0.088, −0.012)	0.010
Model 3	0.031 (0.015)	−0.023 (0.012)	−0.054 (−0.093, −0.015)	0.007
Model 4	0.030 (0.015)	−0.023 (0.012)	−0.052 (−0.092, −0.013)	0.010
Model 5	0.026 (0.016)	−0.020 (0.012)	−0.047 (−0.088, −0.005)	0.027
Model 6	0.027 (0.016)	−0.021 (0.013)	−0.048 (−0.090, −0.006)	0.027
Right maximum IMT-CCA (mean change from baseline; SE)				
Model 1	0.041 (0.035)	−0.007 (0.029)	−0.048 (−0.139, 0.043)	0.30
Model 2	0.039 (0.036)	−0.006 (0.029)	−0.046 (−0.138, 0.046)	0.33
Model 3	0.042 (0.036)	−0.007 (0.029)	−0.049 (−0.143, 0.046)	0.31
Model 4	0.042 (0.036)	−0.007 (0.029)	−0.049 (−0.143, 0.046)	0.31
Model 5	0.045 (0.037)	−0.009 (0.030)	−0.054 (−0.153, 0.045)	0.28
Model 6	0.049 (0.037)	−0.012 (0.030)	−0.061 (−0.160, 0.038)	0.22
Left maximum IMT-CCA (mean change from baseline; SE)				
Model 1	0.027 (0.029)	−0.053 (0.024)	−0.081 (−0.155, −0.007)	0.032
Model 2	0.032 (0.029)	−0.057 (0.024)	−0.089 (−0.164, −0.014)	0.020
Model 3	0.036 (0.029)	−0.057 (0.024)	−0.093 (−0.169, −0.016)	0.017
Model 4	0.033 (0.029)	−0.055 (0.024)	−0.088 (−0.164, −0.012)	0.024
Model 5	0.029 (0.030)	−0.053 (0.024)	−0.082 (−0.161, −0.002)	0.045
Model 6	0.024 (0.031)	−0.049 (0.024)	−0.074 (−0.155, 0.008)	0.075

Differences in delta change in IMT from baseline between two groups were analyzed with analysis of covariance models that included the presence of hypoglycemia, age, gender, baseline IMT and the original treatment group (Model 1), model 1 plus body mass index and current smoking (Model 2), model 2 plus HbA1c, total cholesterol, high density lipoprotein-cholesterol, triglyceride and systolic blood pressure (Model 3), model 3 plus estimated glomerular filtration rate, use of angiotensin-converting enzyme inhibitors/angiotensin II receptor blockers, use of statins and use of anti-platelets (Model 4), model 4 plus the use of sulfonylurea, the use of glinides and the use of α-glucosidase inhibitors (Model 5), model 5 plus C-peptide, high-sensitivity C-reactive protein and interleukin (Model 6). CCA, common carotid artery; IMT, intima-media thickness.

**Table 3:** Changes in IMT from baseline at 104 weeks in patients treated with or without sitagliptin in subgroup analysis based on the occurrence of hypoglycemia.

Parameters	Hypoglycemia	Sitagliptin group	Conventional group	P value
Change in mean IMT-CCA	No	−0.057 ± 0.140 (n = 72)^§^	0.012 ± 0.138 (n = 73)	0.003
	Yes	0.012 ± 0.201 (n = 49)	0.042 ± 0.135 (n = 49)*	0.38
Change in right max IMT-CCA	No	−0.039 ± 0.243 (n = 72)	0.041 ± 0.412 (n = 73)	0.16
	Yes	0.062 ± 0.470 (n = 48)	−0.007 ± 0.295 (n = 49)	0.39
Change in left max IMT-CCA	No	−0.105 ± 0.298 (n = 72)^#^	−0.003 ± 0.304 (n = 73)	0.042
	Yes	−0.005 ± 0.393 (n = 49)	0.060 ± 0.266 (n = 49)	0.34

Data are mean ± SD. Differences in parameters from baseline to 104 weeks within group were analyzed by one-sample t-test. Differences in parameters from baseline to 104 weeks between groups were analyzed by the Student’s t-test. *P < 0.05, ^#^P < 0.01, ^§^P < 0.001.

**Table 4:** Trend associations across groups divided by frequency of hypoglycemia and changes in IMT.

Variable	Regression coefficient (95% Confidence Interval)	P value
Change in mean IMT-CCA		
Model 1	0.047 (0.019–0.075)	<0.001
Model 2	0.048 (0.021–0.075)	<0.001
Model 3	0.043 (0.016–0.070)	0.002
Model 4	0.050 (0.012–0.088)	0.01
Model 5	0.054 (0.015–0.093)	0.007
Model 6	0.052 (0.013–0.092)	0.01
Model 7	0.047 (0.005–0.088)	0.027
Model 8	0.044 (0.014–0.074)	0.005
Change in right max IMT-CCA		
Model 1	0.052 (−0.014–0.118)	0.12
Model 2	0.064 (0.001–0.128)	0.045
Model 3	0.058 (−0.005–0.122)	0.073
Model 4	0.046 (−0.046–0.138)	0.33
Model 5	0.049 (−0.046–0.143)	0.31
Model 6	0.049 (−0.046–0.143)	0.31
Model 7	0.054 (−0.045–0.153)	0.28
Model 8	0.069 (−0.002–0.139)	0.058
Change in left max IMT-CCA		
Model 1	0.077 (0.019–0.134)	0.009
Model 2	0.078 (0.026–0.130)	0.004
Model 3	0.070 (0.018–0.123)	0.009
Model 4	0.089 (0.014–0.164)	0.02
Model 5	0.093 (0.016–0.169)	0.017
Model 6	0.088 (0.012–0.164)	0.024
Model 7	0.082 (0.002–0.161)	0.045
Model 8	0.069 (0.011–0.127)	0.021

Model 1: Trend estimation for linear trends across quintiles is based on linear regression analysis for continuous variables unadjusted (Model 1), adjusted for model 1 plus baseline IMT (Model 2), model 2 plus age, gender, baseline IMT and the original treatment group (Model 3), model 2 plus body mass index and current smoking (Model 4), model 4 plus HbA1c, total cholesterol, high density lipoprotein-cholesterol, triglyceride and systolic blood pressure (Model 5), model 4 plus estimated glomerular filtration rate, use of angiotensin-converting enzyme inhibitors/angiotensin II receptor blockers, use of statins and use of anti-platelets (Model 6), model 6 plus the use of sulfonylurea, the use of glinides and the use of α-glucosidase inhibitors (Model 7), model 7 plus C-peptide, high-sensitivity C-reactive protein and interleukin (Model 8). CCA, common carotid artery; IMT, intima-media thickness.
